# Case Report: Balloon-assisted catheter advancement for antegrade recanalization of non-acute carotid artery occlusions

**DOI:** 10.3389/fsurg.2026.1798320

**Published:** 2026-08-04

**Authors:** Yufeng Wang, Yixin Sun, Ruilin Ren, Lei Xi, Tao Cheng, Yangjun Han, Rong Zhao, Ning Ma, Xinjuan Xu

**Affiliations:** 1 Department of Neurosurgery, The Affiliated Cardiovascular Hospital of Shanxi Medical University and Shanxi Cardiovascular Hospital, Shanxi, China; 2 Department of Hepatobiliary and Pancreatic Surgery, Peking University First Hospital, Beijing, China; 3 Department of Neurosurgery, Xuanwu Hospital, China International Neuroscience Institute, Capital Medical University, National Center for Neurological Disorders, Beijing, China; 4 Department of Neurology, The Affiliated Cardiovascular Hospital of Shanxi Medical University and Shanxi Cardiovascular Hospital, Shanxi, China

**Keywords:** BACAT, endovascular recanalization, hybrid surgery, non-acute internal carotid artery occlusion, open surgery

## Abstract

**Background:**

Endovascular recanalization of non-acute internal carotid artery occlusion (NA-ICAO) remains technically challenging. This technical report describes the Balloon-Assisted Catheter Advancement (BACAT) technique and evaluates its procedural feasibility in a small, heterogenous patient cohort.

**Methods:**

We retrospectively reviewed 15 patients with NA-ICAO who underwent BACAT-assisted recanalization. Cases included both pure endovascular therapy (*n* = 8) and hybrid therapy combined open surgery with endovascualr therapy (*n* = 7). The primary outcome was technical success, defined as successful antegrade passage of the catheter across the occlusive segment using the BACAT maneuver. Procedural complications were recorded descriptively.

**Results:**

Technical success was achieved in 13 of 15 patients (86.7%). No intraprocedural rupture or flow-limiting dissection occurred. One non-disabling postoperative stroke (6.7%) was observed in the hybrid subgroup. Given the small sample, absence of a control group, and case heterogeneity, no statistical comparisons or efficacy claims are made.

**Conclusions:**

The BACAT technique appears technically feasible for selected patients with NA-ICAO, including both pure endovascular therapy and hybrid surgical. This report provides procedural details to facilitate replication and further investigation. Clinical efficacy and comparative safety require validation in larger, controlled studies.

## 1 Introduction

Symptomatic non-acute internal carotid artery occlusion (NA-ICAO) has an estimated annual incidence of 6 per 100,000 individuals (1). Current treatment options include optimal medical management, endarterectomy, and extracranial-intracranial bypass, endovascular recanalization, and hybrid surgery (2–5). However, the optimal management strategy for NA-ICAO remains controversial. A meta-analysis of 22 studies revealed that approximately 20% of patients managed with optimal medical therapy alone faced the higher risk of middle and long-term stroke and mortality (6). Previous studies have showed that extracranial-intracranial bypass does not significantly reduced the risk of the composite outcome of stroke or mortality within 30 days or ipsilateral ischemic stroke beyond 30 days through 2 years (5, 7, 8).

While endarterectomy can directly recanalize stenotic or occluded carotid arteries to improve intracranial perfusion, it is only feasible for cases with shorter occluded ICA segments, with success rates ranging from 40.7% to 87.5% (9). Hybrid surgery offers the dual benefit of plaque removal at the ICA origin and recanalization of long-segment occlusions (10). Endovascular treatment is currently considered the mainstream approach for recanalizing chronic ICA occlusion. Therefore, we introduced the BACAT technique and its application in 15 cases of non-acute ICA occlusion.

## 2 Methods

This study analyzed data from consecutive patients treated with the BACAT technique at our institution. Consecutive patients with NA-ICAO were enrolled from August 2022 to August 2023. Non-acute was defined as subacute (≥24 h to 3 weeks) or chronic (>3 weeks) phase after symptom onset. Our study was approved by the Institutional Review Board of the Shanxi cardiovascular hospital. All patient provided consent for the procedure. The participants and any identifiable individuals consented to publication of his/her image. This study was conducted in accordance with the Dec-laration of Helsinki.

### 2.1 The BACAT technique

The BACAT technique at endovascular recanalization was performed as follows:

1. Pathway establishment: An 8F guiding catheter with an inner 5F or 6F intermediate catheter is placed at the beginning of the ICA or the end of the common carotid artery (Figure 1a);
2. During the procedure, the use of guidewires is as follows: below the proximal cavernous sinus, a V18 guidewire and Rebar18 microcatheter are conventionally applied to reach the distal cavernous sinus. Once reached, a 200 cm neuroguidewire is substituted. While crossing the occlusion, the microguidewire is rotated and its advancement smoothness is continuously assessed to confirm an intraluminal trajectory. Resistance to guidewire passage is interpreted as a sign of subintimal entry, at which point the microguidewire is withdrawn and redirected into the true lumen. Once the microguidewire and microcatheter have traversed the occlusion, blood is aspirated through the microcatheter before slow hand-injection of contrast to reconfirm intraluminal position and assess the distal vasculature (Figure 1b). Under the roadmap of the microcatheter, a 300 cm microguidewire was inserted and advanced to the M2 segment, after which the microcatheter was removed (Figure 1c);
3. Balloon predilation and progressive advancement with an intermediate catheter: A 2.0–20 mm balloon (a 2.0–8.0 mm balloon is used for the cavernous sinus segment and beyond) is advanced from the proximal to the distal end of the cavernous sinus segment, in conjunction with a 5F/125 cm (Cordis) or 6F/125 cm (Navien, Medtronic) intermediate catheter. The balloon is dilation for approximately 20–30 s to ensure the stability of the plaque or thrombus, following which it is slowly deflated to a partially deflated state (Figure 1d). Concurrently, the intermediate catheter is advanced to the distal marker of the balloon (Figures 1e,f). This process of “balloon dilation–semi-deflation—intermediate catheter progressive advancement” is repeated in a stepwise manner up to the distal end of the occlusion segment (Figure 1g). Subsequently, the intermediate catheter is withdrawn to prevent any potential release of thrombus or plaque debris into the intracranial space, thereby mitigating the risk of embolic events;
4. Stent Implantation: With the intermediate catheter in a high-position state, an appropriately sized balloon-expandable or self-expandable stent is selected based on the measured data and implanted sequentially from the distal to the proximal end (Figure 1h). Once accurate stent placement is confirmed, the intermediate catheter is retracted under fluoroscopic guidance from the distal end of the stent to its proximal marker. It is crucial to maintain the intermediate catheter in close proximity to the stent proximal marker, ensuring continuous irrigation to deter thrombus formation within the stent. This also helps to stabilize any temporary thrombus and plaque in the carotid artery segment that has not yet been covered by the stent.
5. Upon reaching a 4 mm diameter with the balloon-expandable stent, it corresponds to the petrous segment of the internal carotid artery. Following stent deployment, the stent balloon may be utilized to dilate the C1 segment of the internal carotid artery, with caution to avoid full expansion at the dissection site. Subsequently, maintaining a 300 cm microguidewire, the 5F or 6F intermediate catheter is extracted, and a self-expanding carotid stent is inserted. In the case of a 6F intermediate catheter, it does not require removal; instead, a self-expanding (Precise, Cordis) carotid stent can be delivered through the 6F intermediate catheter;

**Figure 1:**
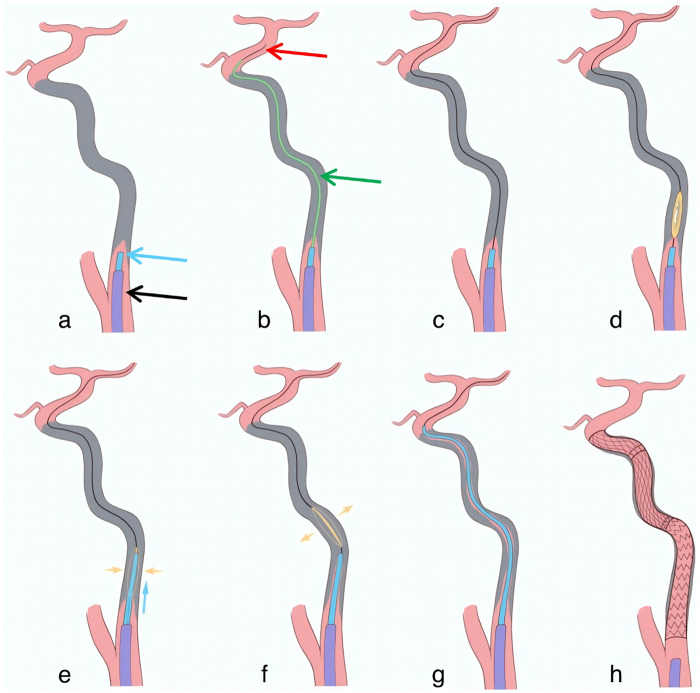
Schematic illustration of the BACAT technique. An 8F guiding catheter (black arrow) with an inner 5F or 6F intermediate catheter (blue arrow) is placed at the beginning of the internal carotid artery (ICA) or the end of the common carotid artery **(a)**; during the procedure, the use of guidewires is as follows: below the proximal cavernous sinus, a V18 microguidewire (red arrow) and Rebar18 microcatheter (green arrow) are conventionally applied to reach the distal cavernous sinus. Upon arrival, a 200 cm neuroguidewire is substituted. After the guidewire crosses the occlusion, it is rotated to observe the movement of its tip to initially confirm the true lumen. The microcatheter was cinfirmed into to the true lumen, and slow hand-injection of contrast is performed to reconfirm the true lumen and assess the distal vasculature **(b)**. Under the roadmap of the microcatheter, a 300 cm microguidewire is exchanged and delivered to the M2 segment, followed by the withdrawal of the microcatheter **(c)**. Balloon (yellow arrow) predilation and progressive advancement with an intermediate catheter: A 2.0–20 mm balloon (a 2.0–8.0 mm balloon is used for the cavernous sinus segment and beyond) is advanced from the proximal to the distal end of the cavernous sinus segment, in conjunction with a 5F/125 cm (Cordis) or 6F/125 cm (Navien, Medtronic) intermediate catheter. The balloon is inflated for approximately 20–30 s to ensure the stability of the plaque or thrombus, then slowly deflated to a semi-deflated state **(d)**. Concurrently, the intermediate catheter is advanced to the distal marker of the balloon **(e,f)**. This process of “balloon inflation—semi-deflation—intermediate catheter progressive advancement” is repeated in a stepwise manner up to the distal end of the occlusion segment **(g)**. The intermediate catheter is then retracted to prevent the escape of any thrombus or plaque debris into the intracranial space, which could lead to embolic events. Stent Implantation: With the intermediate catheter in a high-position state, an appropriately sized balloon-expandable or self-expandable stent is selected based on the measured data and implanted sequentially from the distal to the proximal end **(h)**.

Hybrid surgery was performed as follows:

During hybrid surgery, after plaque and thrombus removal, the catheter and guidewire are precisely advanced into the true lumen under visual guidance. The decision to perform vessel suturing was guided by the morphology of the distal ICA stump as observed during endarterectomy. Following the completion of endovascular recanalization, the sheath was extracted, and the incision was closed subcutaneously and cutaneously. To mitigate the risk of intimal disruption, partial expansion only was permitted at the endarterectomy site.

To standardize outcome definitions and follow-up, the primary efficacy endpoint was defined as in-stent restenosis (≥50% luminal narrowing) on follow-up imaging (computed tomography angiography, CTA, or MR angiography), and the primary safety endpoint was any ischemic or hemorrhagic stroke within 30 days. Routine diffusion weighted imaging (DWI) was performed on postoperative day 1 in all patients to detect procedure-related ischemic lesions; the number and proportion of patients completing this examination are reported. Restenosis was monitored longitudinally by CTA or MR angiography at 3 and 6 months. Whenever new neurological symptoms emerged after recanalization, urgent brain CT or MRI was performed. The overall follow-up duration was at least 3 months.

### 2.2 Periprocedural management

All patients were receiving clopidogrel 75 mg/day and aspirin 100 mg/day for at least 5 days before the procedure. The surgical interventions included hybrid surgery and endovascular recanalization, which were performed under general anesthesia. During the procedure, unfractionated heparin was administered intravenously at an initial bolus of 70 U/kg after arterial sheath placement, followed by additional boluses of 1,000 U every hour to maintain an activated clotting time (ACT) between 250 and 300 s. Transcranial oximetry was not available; reperfusion was assessed by immediate post-procedural CTP. Cerebrovascular perfusion examination computed tomography perfusion (CTP) showed hypoperfusion of cerebral hemisphere on the ipsilateral of the target artery. Subsequent to the procedure, patients continued the dual antiplatelet therapy with clopidogrel 75 mg and aspirin 100 mg for either 3 or 6 months, followed by lifelong monotherapy with antiplatelet agents.

## 3 Results

A total of 15 patients (all male; mean age 59.6 ± 9.8 years) were included. The baseline comorbidities were hypertension (14/15, 93.3%), previous stroke or transient ischemic attack (7/15, 46.7%), peripheral artery disease (5/15, 33.3%), diabetes mellitus (4/15, 26.7%), hyperlipidemia (4/15, 26.7%), and coronary artery disease (1/15, 7.0%). Endovascular therapy alone was performed in 7 patients (46.7%), and hybrid surgery in 8 patients (53.3%). Technical success was achieved in all 15 patients (100%). Over a mean follow-up of 8.1 ± 3.4 months, the imaging follow-up completion rate was 100% (15/15). No postoperative hemorrhage or 30-day stroke occurred. At the last follow-up, a favorable functional outcome (modified Rankin Scale 0–3) was achieved in 14 patients (93.3%), and in-stent restenosis was observed in 2 patients (13.3%). A summary of the main baseline and outcome data is shown in Table 1. The detailed clinical characteristics, imaging results, and follow-up data for all 15 patients are presented in Table 2.

**Table 1:** Summary of baseline characteristics and outcomes.

Variable	Value
Age, years, mean ± SD	59.6 ± 9.8
Male, *n* (%)	15 (100%)
Previous stroke or TIA, *n* (%)	7 (46.7%)
Hypertension, *n* (%)	14 (93.3%)
Diabetes mellitus, *n* (%)	4 (26.7%)
Hyperlipidemia, *n* (%)	4 (26.7%)
Coronary artery disease, *n* (%)	1 (7%)
Peripheral artery disease, *n* (%)	5 (33%)
Endovascular therapy, *n* (%)	7 (46.7%)
Hybrid surgery, *n* (%)	8 (53.33%)
Technical success, *n* (%)	15 (100%)
Follow-up duration (month), mean ± SD	8.06 ± 3.38
Outcomes	
Postoperative hemorrhage, *n* (%)	0
Any stroke within 30 days, *n* (%)	0
Imaging follow-up completion rate, *n* (%)	15 (100%)
mRS 0–3 at last follow-up, *n* (%)	14 (93.3%)
In-stent restenosis, *n* (%)	2 (13.3%)

TIA, transient ischemic attack; mRS, modified Rankin Scale; SD, standard deviation. Values are presented as number (%), mean ± SD. Percentages may not total 100% due to rounding.

**Table 2:** Demographic and clinical characteristics of patients with BACAT technique.

Characteristic	Sex/Age	Pre mRS	Progressive stroke	Previou stroke or TIA	Symptoms on admission	Comorbidities	Infarct location on head imaging	Treatment	Complications of hospitalization	Restenosis	Post TICI	Post mRS
Patient 1	Man/58	4	No	None	Hemispheric ischemic event	Hypertension, DM	NA-acute cerebral infarction in the right insular, frontal, temporal and parietal lobes	CAS	None	None	3	0
Patient 2	Man/64	5	Yes	Stroke	Hemispheric ischemic event	Hypertension, DM, PVD	NA-acute cerebral infarction in the left temporal and frontal-parietal lobe.	CAS	None	None	3	3
Patient 3	Man/50	3	No	None	Hemispheric ischemic event	Hypertension, DM, hyperlipidemia	NA-acute cerebral infarction in the left temporal	CAS	None	None	3	0
Patient 4	Man/48	5	No	Stroke	None	Hypertension, DM, hyperlipidemia, PVD	Multiple old cerebral infarctions occurred in the bilateral basal ganglia region, the right periventricular area, and the frontal-parietal lobes.	CAS	None	None	3	4
Patient 5	Male/69	0	No	None	None	Hypertension, DM, PVD	NA-acute cerebral infarction in the right lateral ventricle, the centrum semiovale, and the parietal lobe	CAS	None	None	3	1
Patient 6	Man/43	3	No	Stroke	Hemispheric ischemic event	Hypertension, DM	Multiple NA-acute cerebral infarctions occurred in the right temporal insular lobe, basal ganglia region, periventricular area of the lateral ventricle, paracentral lobule, and frontal lobe	CAS	None	None	3	2
Patient 7	Man/64	2	No	None	Hemispheric ischemic event	Hypertension	NA-acute cerebral infarction in the left lateral ventricle parietal region, the centrum semiovale, and the subcortical area of the frontal lobe	Hybrid surgery	None	None	3	0
Patient 8	Man/64	2	No	None	Hemispheric ischemic event	Hypertension, coronary heart disease, PVD	Multiple old lesions in the right cerebral hemisphere	CAS	None	None	3	1
Patient 9	Man/53	0	Yes	Stroke	Hemispheric ischemic event	Hypertension, hyperlipidemia, PVD	Multiple old lesions in the left cerebral hemisphere	Hybrid surgery	None	None	3	1
Patient 10	Man/59	1	No	None	None	Hypertension, DM	NA-acute stage cerebral infarction in the right hemispheric center of the cerebellum	Hybrid surgery	None	None	3	0
Patient 11	Man/69	2	No	Stroke	None	Hypertension, hyperlipidemia	Multiple old lesions in the left cerebral hemisphere	Hybrid surgery	None	Yes	3	0
Patient 12	Man/73	0	No	TIA	None	Hypertension, DM	None	Hybrid surgery	None	Yes	3	0
Patient 13	Man/63	3	No	Stroke	None	Hypertension, hyperlipidemia, PVD	Multiple old lesions in the left cerebral hemisphere	Hybrid surgery	None	None	3	2
Patient 14	Man/45	2	No	None	None	Hypertension	Left lateral ventricle periphery, parahippocampal region, and frontal lobe infarction lesion	Hybrid surgery	None	None	3	0
Patient 15	Man/72	5	No	None	Hemispheric ischemic event	No	Left temporal insular lobe, periventricular region of the lateral ventricle, and paracentral lobule infarction	Hybrid surgery	None	None	3	3

DM, diabetes mellitus; PVD, peripheral vascular artery; TIA, transient ischaemic attack, non-acute is defined as subacute or chronic cerebral infarction.

## Case presentation

A 63-year-old patient with right-sided limb numbness, weakness, and fluency disorder was transferred to our hospital with a diagnosis of no-acute ischemic stroke. Head CT and MRI revealed infarction in the left frontal and temporal lobes (Figures 2A,B). DSA showed left ICAO (Figures 3A,B). Endovascular recanalization of the left ICA was performed (Figures 3C–V). Follow-up DSA confirmed successful recanalization, and no further events occurred at 1-year (Figures 3Y,Z).

**Figure 2:**
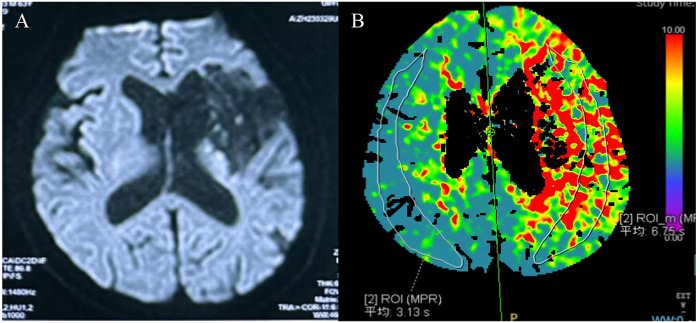
**(A)** Preoperative DWI shows infarction in the left frontal and temporal lobes; **(B)** CTP imaging with a left MTT of 6.75 s.

**Figure 3:**
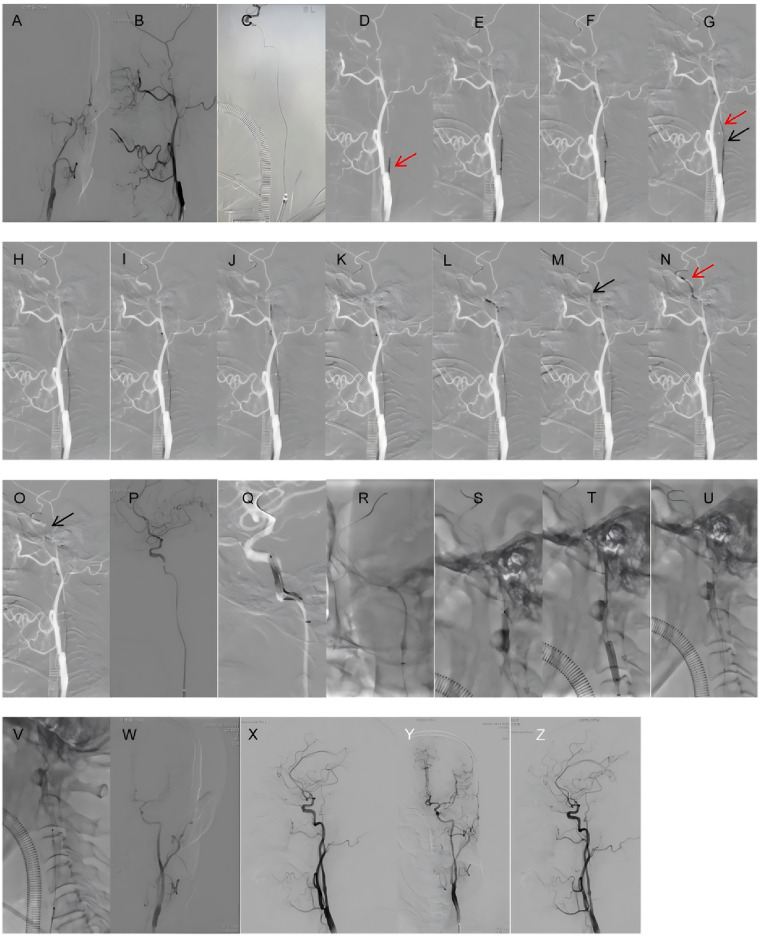
Case example. **(A,B)** DSA showed left internal carotid artery chronic occlusion; **(C)** Microguidewire successfully traversing the occluded segment to reach the communicating segment of internal carotid artery; **(D–O)** balloon dilation from the cervical segment to the ophthalmic segment, performed with the assistance of a middle catheter. (black arrow indicates the tip position of the middle catheter, the red arrow marks the distal end of the balloon). **(P)** Angiographic imagine after balloon dilation; **(Q–V)** The sequential stent placement from the distal segment (ophthalmic artery segment) to the proximal segment (cervical segment), using the following stents: MicroPort Firbird 3 × 29, Apollo 3.5 × 8, MicroPort Firbird 4 × 29, Yalen drug-eluting stent 4.5 × 16, Cordis Precise 6 × 40, and Cordis Precise 8 × 40. **(W,X)** Postoperative angiographic images. **(Y,Z)** DSA showed patent of left carotid artery after the 1-year.

## 4 Discussion

In the present study, we assessed the safety and efficacy of a novel endovascular recanalization technique—the BACAT technique—in patients with NA-ICAO. Our results demonstrated that, in the BACAT group, the technical success rate was 100% and the periprocedural complication rate was 0%.

The treatment modalities for NA-ICAO encompass medical treatment, and cerebral revascularization. Multiple studies have confirmed that cerebral revascularization is technically feasible, with reported success rate ranging from 60% to 100% (11–14). Moreover, revascularization has been shown to not only reduce intraoperative complications but also improve overall procedural efficacy (15). However, these studies primarily focused on NA-acute occlusions, and evidence specific to the NA-ICAO population remains limited.

It serves three key functions: (1) establishing stable access to the proximal end of the target lesion; (2) stabilizing thrombi and plaques to minimize distal embolization; and (3) providing a robust platform for the advancement of microcatheters, microwires, and subsequent devices. This technical advantage is supported by comparative evidence. For instance, Zhai et al., reported that micro-guidewires could not traverse the origin of the occluded ICA and entered the distal true lumen despite repeated attempts (16). In contrast, Cheng et al. demonstrated that PEACE technique, which employs an intermediate catheter to navigate extracranial ICA lesions, significantly reduced the time from femoral access to recanalization and improved the success rate in patients with extracranial ICA occlusion (17).

Preventing embolization is crucial during any recanalization procedure due to the presence of intraluminal thrombi. A recent meta-analysis reported that proximal balloon protection devices reduce the risk of ischemic stroke in patients undergoing carotid artery stenting (18). However, the use of such protection devices can be challenging in patients with insufficient space within the distal true lumen. In our study, the BACAT technique, combined with the intermediate catheter, functioned as a large lumen suction catheter to remove thrombi or plaque debris under continuous blood flow. Notably, no new post-procedural DWI lesions were identified in patients treated with the BACAT technique.

Atherosclerosis and vessel tortuosity often pose significant challenges in accessing the target lesion. Previous studies have shown that the use of intermediate catheters with a coaxial system reduces instrument exchanges during surgery, provides stable support, and lowers the incidence of intraoperative complications such as vasospasm, intimal dissection, hemorrhage or cavernous sinus fistulas (19, 20). Chen et al. reported that this technique enhanced the endovascular recanalization success rate to 89.8%, while reducing complications (21), which is consistent with our findings. In the present study, no intraoperative complications were observed with the BACAT technique. Nevertheless, given the limited sample size, further research is required to more comprehensively elucidate the relationship between the BACAT technique and the reduction of intraoperative complications.

Despite successful recanalization of CICAO, the rates of restenosis and reocclusion remain relatively high. Multiple studies have reported rates range from 10% to 43% following the procedure (11, 12, 22). Lu et al. demonstrated a cumulative restenosis rate of 42.3% at 48-month follow-up after revascularization for non-acute ICAO (23). In our study, the BACAT group showed a more favorable outcome, with only two cases of restenosis and no reocclusion.

The BACAT technique appears to offers significant advantages over traditional endovascular treatments, including improved success rates in navigating occlusions, reduced embolic risks, and precise stent placement. However, due to the small sample size and retrospective nature of this study—acknowledging that the data were derived from a prospectively maintained institutional database—further prospective studies with larger cohorts are needed to confirm its long-term safety and efficacy. Furthermore, while patients with no-acute occlusion and established neurological deficits may not experience immediate clinical improvement, recanalization may still confer benefits by improving hemodynamic reserve and potentially reducing the risk of future ipsilateral ischemic events.

## 5 Conclusion

This case series of 15 heterogenous NA-ICAO suggests that the BACAT thchnique is technically feasibal with acceptable short-term outcomes. No definitive claims regarding functional efficacy or superiority over other recanalization strategies can be made from this non-comparative, small-sample experience. Future investigations with larger, controlled cohorts are needed to refine the technique, establish procedural safety with greater certainty, and evaluate long-term outcomes.

## Data Availability

The raw data supporting the conclusions of this article will be made available by the authors, without undue reservation.
